# Health resource utilization, costs, and community treatment order status before and after the initiation of second-generation long acting-injectable antipsychotics in patients with schizophrenia in Alberta, Canada

**DOI:** 10.1192/j.eurpsy.2022.326

**Published:** 2022-09-01

**Authors:** P. Chue, K.O. Wong, S. Klarenbach, K. Martins, S. Dursun, M. Snaterse, L. Richer

**Affiliations:** UNIVERSITY OF ALBERTA, Psychiatry, EDMONTON, Canada

**Keywords:** LONG ACTING ANTIPSYCHOTIC INJECTION, HEALTH RESOURCE UTILIZATION, COMMUNITY TREATMENT ORDER, schizophrénia

## Abstract

**Introduction:**

Long-acting injectable (LAI) antipsychotics and community treatment orders (CTOs) are used in patients with schizophrenia to improve treatment effectiveness through adherence.

**Objectives:**

Understanding healthcare resource utilization (HRU) and associated costs, and medication adherence in patients with schizophrenia overall and by CTO status before and after second generation antipsychotic (SGA) LAI initiation may guide strategies to optimize health.

**Methods:**

A retrospective observational single-arm study using administrative data from Alberta was performed. Adults with schizophrenia who initiated SGA-LAI (index date) were included. Medication possession ratio (MPR) was determined; paired t-tests were used to examine differences in HRU and costs ($CDN) between the 2-year pre-index period and 2-year post-index period. Stratified analysis by presence or absence of an active CTO during the pre-post periods was performed.

**Results:**

Among 1,211 patients who initiated SGA-LAIs, MPR was greater post-index (0.84) compared with pre-index (0.45; 95% confidence interval [CI] 0.36, 0.41). All-cause and mental health-related HRU and costs were lower post-index versus pre-index (p<0.001); total all-cause HRU costs were $33,788 lower post- versus pre-index ($40,343 [standard deviation, SD $68,887] versus $74,131 [SD $75,941], 95% CI [-$38,993, -$28,583]), and total mental health-related HRU costs were $34,198 lower post- versus pre-index ($34,205 [SD $63,428] CDN versus $68,403 [SD $72,088] CDN, 95%CI [-$39,098, -$29,297]). Forty-three percent had ≥1 active CTO during the study period; HRU and costs varied according to CTO status.

**Conclusions:**

SGA-LAIs are associated with improved adherence, and lower HRU and costs however the latter vary according to CTO status.

**Disclosure:**

The author(s) declared the following potential conflicts of interest with respect to the research, authorship, and/or publication of this abstract: SD and MS have no competing interest to declare. LR, SK, KW, and KM are members of the Real-World Evidence

